# The relative contributions of speechreading and vocabulary to deaf and hearing children's reading ability

**DOI:** 10.1016/j.ridd.2015.10.004

**Published:** 2016-01

**Authors:** Fiona Elizabeth Kyle, Ruth Campbell, Mairéad MacSweeney

**Affiliations:** a Deafness, Cognition and Language Research Centre (DCAL), Division of Psychology and Language Sciences, UCL, London, UK; b Institute of Cognitive Neuroscience, UCL, London, UK

**Keywords:** Deaf, Reading, Speechreading, Vocabulary

## Abstract

**Background:**

Vocabulary knowledge and speechreading are important for deaf children's reading development but it is unknown whether they are independent predictors of reading ability.

**Aims:**

This study investigated the relationships between reading, speechreading and vocabulary in a large cohort of deaf and hearing children aged 5 to 14 years.

**Methods and procedures:**

86 severely and profoundly deaf children and 91 hearing children participated in this study. All children completed assessments of reading comprehension, word reading accuracy, speechreading and vocabulary.

**Outcomes and results:**

Regression analyses showed that vocabulary and speechreading accounted for unique variance in both reading accuracy and comprehension for deaf children. For hearing children, vocabulary was an independent predictor of both reading accuracy and comprehension skills but speechreading only accounted for unique variance in reading accuracy.

**Conclusions and implications:**

Speechreading and vocabulary are important for reading development in deaf children. The results are interpreted within the Simple View of Reading framework and the theoretical implications for deaf children's reading are discussed.

## Highlights

• Speechreading predicted both reading accuracy and comprehension in deaf children.
• Speechreading predicted only reading accuracy in hearing children.
• Vocabulary was strongest predictor of reading in both deaf and hearing children.
• Deaf children who preferred to communicate through speech were better speechreaders.
• Findings were discussed with reference to the Simple View of Reading.

## 1 Introduction

Despite having intelligence scores in the normal range, the majority of deaf children have poorer reading outcomes than their hearing peers (e.g. Conrad, 1979, Kyle and Harris, 2010, Lederberg et al., 2013, Wauters et al., 2006). Large scale studies report that deaf school leavers have reading ages far behind their chronological ages (see Qi & Mitchell, 2011 for a review) and reading skills seem to develop at only a third of the rate of hearing children (Allen, 1986, Kyle and Harris, 2010). There is a consistent picture of underachievement in reading skills which can have long-lasting effects upon future employment opportunities. It is therefore imperative to gain a better understanding of which cognitive and language skills are important for reading development in deaf children and the complex relationships between these abilities. Recent research has suggested that speechreading (silent lipreading) and vocabulary are longitudinal predictors of deaf children's reading development (Kyle and Harris, 2010, Kyle and Harris, 2011), and that speechreading is also predictive of reading ability in hearing children (Kyle & Harris, 2011). However, the relative contribution of these two skills to reading is unknown; therefore the main aim of this study is to examine whether speechreading and vocabulary are independent predictors of reading in deaf and in hearing children.

The predictors of reading ability, and the often complex relationships between predictors, are well documented in hearing children. One of the most widely-acknowledged predictors of early reading is phonological knowledge and skills (e.g. Adams, 1990, Castles and Coltheart, 2004). Children with better phonological awareness (the ability to detect and manipulate the constituent sounds of words) and greater knowledge about the relationships between letters and sounds tend to make the most progress in reading in the early stages (see Castles and Coltheart, 2004, Goswami and Bryant, 1990). However, it is also well known that different cognitive and language based skills are predictive of different components of the reading process, i.e. letter-sound knowledge and phonological skills are most predictive of word recognition and word reading whereas higher order language skills such as grammar and syntax are most predictive of reading comprehension (see Catts and Weismer, 2006, Muter et al., 2004, Oakhill et al., 2003, Storch and Whitehurst, 2002). Vocabulary is generally thought of as being most important in the beginning stages of reading where it predicts initial word recognition (e.g. Dickinson et al., 2003, Verhoeven et al., 2011) and emerging comprehension skills (e.g. Anderson and Freebody, 1981, Ricketts et al., 2007, Roth et al., 2002); however, research suggests that vocabulary knowledge also plays an important role in later reading skills (e.g. Senechal et al., 2006, Verhoeven et al., 2011).

The question of whether phonological skills are important, or even necessary, for deaf children's reading skills is a matter of ongoing debate. In summary, the research evidence is very mixed. Some authors find evidence for the role of phonological skills in deaf children's reading (e.g. Campbell and Wright, 1988, Dyer et al., 2003, Easterbrooks et al., 2008) while many others report a very small or non-significant relation (e.g., Hanson and Fowler, 1987, Kyle and Harris, 2006, Leybaert and Alegria, 1993, Mayberry et al., 2011, Miller, 1997). These discrepancies hold true even when traditional phonological awareness assessments are adapted to make them more deaf-friendly, i.e. by representing the items pictorially. A recent meta-analysis of the literature concluded that there was little evidence that deaf individuals use phonology in their reading (Mayberry et al., 2011). In their analyses Mayberry et al. (2011) included 25 studies that looked at the relation between reading and phonological coding and awareness in deaf individuals, ranging from young children to adults and from across the spectrum of language and communication preferences (sign/speech). The resulting effect sizes for the relationship between reading and phonological awareness ranged from −.13 to .81 with a mean of .35. This means that on average, across the 25 studies, 11% of the variance in reading skills in deaf participants was explained by phonological abilities. This figure refers to the contribution of spoken phonology to reading for deaf participants. It should be noted that signed languages are also phonologically structured, albeit with different parameters (e.g. Brentari, 1999, Sandler and Lillo-Martin, 2006). Corina, Hafer, and Welch (2014) recently reported a positive correlation between phonological awareness of American Sign Language and phonological awareness of English. Furthermore, McQuarrie and Abbott (2013) reported a correlation of .47 between phonological awareness in American Sign Language (ASL) and reading. However, as yet there is no evidence for a direct causal relationship between the knowledge of sign language phonology and reading.

An important caveat regarding the Mayberry et al. meta-analysis is that the study mainly included correlational studies. Due to the low number of longitudinal studies in deaf children only two such studies were included (Harris and Beech, 1998, Ormel, 2008). Correlational relationships between variables do not infer causality. Furthermore, in longitudinal studies of reading development it is important to control for early levels of reading ability on later reading outcomes because of the well documented auto-regressor effect, whereby the strongest predictor of later ability is typically earlier achievement in that particular skill (see Caravolas et al., 2001, Castles and Coltheart, 2004). In the only two longitudinal studies of deaf reading development that have used this approach, the data suggest that deaf children may develop their phonological skills through reading (Kyle and Harris, 2010, Kyle and Harris, 2011). Reading and phonological awareness were found to be related in deaf children, but the direction of the observed relation was from earlier reading ability to later phonological awareness. Therefore this may be different to the typical relation seen in hearing children whereby phonological awareness is a strong initial predictor of reading ability and then the two skills tend to exhibit a reciprocal relation (Burgess and Lonigan, 1998, Castles and Coltheart, 2004). In deaf children, it seems that it is reading ability that initially predicts phonological awareness, but then, similar to hearing children, the two skills become reciprocally related and develop in a common and mutually beneficial manner. This pattern of development also fits in with an emerging pattern in the deaf literature, that of the predictive relation between phonological awareness and reading being stronger, or more apparent, in older deaf children and adults, than in young children. That is, the role of phonological skills in deaf reading becomes stronger once reading skills are more proficient. It is important to note that the studies included in the meta-analysis of reading and phonological awareness studies conducted by Mayberry et al. (2011) covered deaf participants from a very broad age range, from early childhood to adulthood. New insights from the longitudinal studies outlined above suggest that combining data across this very wide age range of deaf participants may not be appropriate.

A different way of looking at whether phonological skills have a role in deaf reading is to examine the contribution of visual-based phonological skills such as lipreading or speechreading. There is growing evidence that some deaf individuals do make use of phonology but that their phonological strategy may be slightly different to that of hearing individuals because it is mainly derived from speechreading information rather than auditory input. Speechreading (or lipreading), which is the skill of processing speech from the visible movements of the head, face and mouth, has the potential to be useful in phonological processing when hearing is absent. In addition to providing visual information about vowels (mouth shape), some consonantal phonemes are ‘easy to see’ and ‘hard to hear’. For example, /n/ and /m/ form one pair of consonants that are confusable auditorily, but clear visually (see Summerfield, 1979).

Individual differences in speechreading skill have been found to predict reading outcomes in deaf individuals, both cross-sectionally and longitudinally (Arnold and Kopsel, 1996, Geers and Moog, 1989, Kyle and Harris, 2006, Kyle and Harris, 2010, Kyle and Harris, 2011). If the information gleaned through speechreading is used as the input for a phonological code, then the better one is at speechreading, the more specified and distinct the underlying representations are likely to be. These underlying representations can then be used to form the basis for a phonological code. The quality of phonological representations is thought to be related to reading ability (Elbro, 1996, Swan and Goswami, 1997) because the more specified and distinct the underlying representations, the better able the individual is to complete phonological awareness tasks (Elbro, Borstrøm, & Petersen, 1998) and performance on these types of tasks is extremely indicative of reading ability (see Castles & Coltheart, 2004 for a review). The strong predictive relationships found between speechreading and reading in deaf children (e.g. Kyle and Harris, 2010, Kyle and Harris, 2011), the presence of speechread errors in both deaf children's spelling (Burden and Campbell, 1994, Leybaert and Alegria, 1995, Sutcliffe et al., 1999) and in their performance on phonological awareness tasks (Hanson et al., 1983, Leybaert and Charlier, 1996) suggest that, when deaf children are learning to read, the better they are at speechreading the more information they will be able to use when making connections between letters and sound (see also Alegria, 1996, Campbell, 1997).

Many of the earlier studies only found speechreading was associated with levels of reading ability in orally educated deaf children (e.g. Arnold and Kopsel, 1996, Campbell and Wright, 1988, Craig, 1964, Geers and Moog, 1989); however, more recent research has reported speechreading to be a strong longitudinal predictor of deaf children's reading development, regardless of language preference (Kyle and Harris, 2010, Kyle and Harris, 2011). This shift can be readily explained by the changes in deaf educational practices in the UK over the past 30 years as fewer deaf children are now educated in specialist schools and there is more integration in mainstream schools. This, combined with the introduction of bilingual education through British Sign Language (BSL) and English, has impacted upon the teaching of reading and language to deaf children making it more likely that almost all of them are exposed to both oral speech and sign to some extent.

The other skill that is increasingly reported as being important for deaf children's reading ability is vocabulary knowledge (e.g. Geers and Moog, 1989, Kyle and Harris, 2006, Kyle and Harris, 2010, Kyle and Harris, 2011, LaSasso and Davey, 1987, Mayberry et al., 2011, Moores and Sweet, 1990). Vocabulary and language skills seem to be imperative for deaf reading regardless of how either skill is assessed or which component of reading is measured. ‘Language skills’ were found to be the largest contributor to reading ability in the Mayberry et al. (2011) meta-analysis. The category ‘language skills’ included measures of signed and spoken vocabulary (amongst other measures). Vocabulary was also the strongest and most consistent longitudinal predictor of both word reading and reading comprehension in the Kyle and Harris, 2010, Kyle and Harris, 2011. This could be considered relatively unsurprising given the well documented language delays in deaf children (Waters and Doehring, 1990, Musselman, 2000). In hearing children, vocabulary and good language skills have been proposed as providing a possible compensatory mechanism for children who have poor phonological skills (e.g. Nation and Snowling, 1998, Snowling et al., 2003). This explanation is equally plausible, if not more so, for the strong relationship between vocabulary and reading in deaf children.

Previous studies looking at the role of vocabulary and speechreading in deaf children's reading have had insufficient sample sizes to determine whether these two skills are independent predictors of reading in deaf children. In Kyle and Harris (2010), speechreading was mainly a longitudinal predictor of early reading skills and thus it is of interest to determine whether this relationship feeds into vocabulary development. It would make sense that deaf children who have better speechreading skills have larger vocabularies yet the converse relationship whereby having a more extensive vocabulary would enable one to be a better speechreader is also likely (Davies, Kidd, & Lander, 2009). What is not known is whether speechreading and vocabulary, which are likely to be related themselves, make independent contributions to reading or whether they are simply reflecting some common underlying language factor or capacity. On the other hand, these two factors could interact in a more complex developmental manner, for example, one skill may ‘jumpstart’ the development of the other skill at one stage, but then become less relevant.

Vocabulary and speechreading underpin the model of deaf reading proposed by Kyle (2015), which was based upon the Simple View of Reading (Gough & Tunmer, 1986). The Simple View of Reading postulates that reading is made up of two components: a decoding component and a linguistic component, both of which are necessary for reading. For deaf children, as argued by Kyle (2015) and Kyle and Harris (2011), speechreading contributes to phonological representations and thus forms the basis, along with phonological awareness, for the decoding component, and vocabulary knowledge contributes to the linguistic component. This is not to say that other skills are unimportant for reading in deaf individuals but the role of these two particular skills is explored in the current study. It should also be noted that the relationship between speechreading and reading has mainly been measured at the level of the single word reading and single word speechreading. It is therefore unknown whether speechreading at different linguistic levels also predicts reading and whether the strength of this relationship varies for different reading components. Kyle and Harris, 2006, Kyle and Harris, 2010 found that single word speechreading was significantly related to word reading but not reading comprehension and the relationship was stronger with word reading than sentence comprehension. The current study uses a recently developed Test of Child Speechreading (ToCS; Kyle, Campbell, Mohammed, Coleman, & MacSweeney, 2013), which assesses speechreading at three different levels: words, sentences and sort stories. We investigate how performance at these different levels is related to word reading and reading comprehension. This will help to shed light upon the role that speechreading plays in reading, for example, is the relationship simply at the lexical level or does it reflect broader linguistic knowledge?

Kyle and Harris (2011) also reported that speechreading of single words was longitudinally predictive of beginning reading development in hearing children. This finding warrants further investigation as although it is easy to understand why speechreading is predictive of reading in deaf individuals, due to impaired auditory access, it may not be immediately obvious why speechreading would also be predictive of reading growth in hearing children. We would argue that a similar explanation also holds for hearing children: speechreading is related to reading because the visual speech information derived through speechreading is likely to be incorporated into phonological representations. Therefore, better speechreading skills may result in more distinct and specified phonological representations which in turn can help children when learning to read. The key difference is the supplementary nature of this information and detail for hearing children. Evidence from research with blind children supports this viewpoint as studies often report delays in discriminating phonological contrasts that are difficult to distinguish in the auditory domain but are visually distinct (Mills, 1987).

Lastly, given the suggested role of speechreading in reading, it is important to understand what makes a good child speechreader. Research with adults has shown that better speechreaders tend to be deaf, use oral language to communicate, have higher levels of reading ability and report they can understand the public (Bernstein, Demorest, & Tucker, 1998). No such comparable studies have been conducted with deaf children but the findings from separate studies do help shed light on possible correlates of speechreading. Relations have previously been reported between speechreading and vocabulary in young hearing children (Davies et al., 2009) and between speechreading and working memory (Lyxell & Holmberg, 2000), phonological awareness (Lyxell and Holmberg, 2000, Kyle and Harris, 2010) and NVIQ (Craig, 1964) in hearing-impaired children. Interestingly, recent research has shown no difference between deaf and hearing children in their speechreading ability (Kyle et al., 2013) but speechreading was found to improve with age (Kyle et al., 2013). Moreover, when using an adult speechreading test very similar to the ToCS, deaf adults were found to have superior speechreading skills in contrast to their hearing peers (Mohammed et al., 2006, Mohammed et al., 2003). The contribution of demographic, background and audiological factors to children's speechreading will be investigated in the current study.

This study explores the role of speechreading and vocabulary in reading ability with a large sample of deaf and hearing children. The main aims were to (1) to determine whether speechreading and vocabulary are independent predictors of reading ability in deaf children; (2) to investigate the role of speechreading at different linguistic levels for different components of reading ability (i.e. does speechreading at different linguistic levels exhibit different relationships with reading components); (3) to examine whether speechreading and vocabulary are independent predictors of reading in hearing children; and (4) to explore the effect of demographic and background variables on speechreading.

## 2 Method

### 2.1 Participants

Eighty-six deaf children and 91 hearing children aged between 5 and 14 years old took part in this study. The mean age of the deaf children was 9 years 6 months (*SD* *=* *31.5*) and the mean age of the hearing children was 9 years 1 month *(SD* *=* *30.2).* Thirty-nine deaf children and 53 hearing children were male. Deaf children were recruited from specialist schools for the deaf and resource bases for students with hearing impairments attached to mainstream schools across Southern England. To ensure that deaf and hearing children were similar in terms of demographic backgrounds, the hearing children were recruited from the mainstream schools to which the resource bases were attached. Deaf and hearing children were from a range of different ethnic backgrounds: 59% of the children were White British or White European, 11% were Black British or Black Other, 21% were Asian British or Asian Other and the remaining 9% were mixed race or other. There were no significant differences between the deaf and hearing children in their gender distribution (*X*^2^(1) = 2.94, ns), ethnicity (*X*^2^(3) = 6.99, ns), chronological age (*t*(175) = .98, ns) and NVIQ (*t*(175) = −1.87, ns).

All deaf children had a severe or profound bilateral hearing loss of greater than 70 db with a mean loss of 97.7 db. Thirty-five of the deaf children had cochlear implants (CI) and the remaining (apart from two) wore digital hearing aids. The average age at which deafness was diagnosed was 17 months (SD = 12.3). The majority of deaf children were in hearing-impaired resource bases attached to mainstream schools but a third were in specialist schools for the deaf. Children varied in their language and communication preferences: 44 preferred to communicate through speech; 33 preferred to use signing (26 used BSL and 7 used Sign Supported English); six used total communication (a mixture of both signing and speech) and the remaining three were bilingual in spoken English and BSL. Table 1 presents descriptive statistics for background information for the deaf participants separated out for device use (CI, digital hearing aids and no device)

### 2.2 Materials

Four tasks were administered to assess reading ability, speechreading skills, expressive vocabulary and NVIQ.

#### 2.2.1 Reading ability

The Neale Analysis of Reading II (NARA II: Neale, 1997) was used to assess reading accuracy and reading comprehension skills. Children were shown a booklet containing short passages and asked to read them aloud in their preferred communication mode: English, BSL or a combination of the two. They were then asked a series of questions about each passage to test their comprehension, which they were allowed to answer in their preferred communication. Children received an accuracy score for their word reading and for their comprehension skills. The task was administered according to the instruction manual, apart from the instructions being delivered in the child's preferred language or communication method.

#### 2.2.2 Speechreading ability

Speechreading ability was measured using the Test of Child Speechreading (ToCS: Kyle et al., 2013). The ToCS is a child-friendly, computer-based assessment that measures silent speechreading at three different psycholinguistic levels: words, sentences and short stories. It uses a video-to-picture matching design whereby children are presented with silent video clips of either a man or a woman speaking and they have to choose the picture (from an array of four containing the target and 3 distractors) that matched what was said in the video clip. For example, in the word subtest, for the target item “door”, the pictures were “door”, “duck”, “fork” and “dog”. An example of a sentence trial was the target “The baby is in the bath” and the distractors were pictures depicting a baby reading a book, some pigs on a path and an elephant having a bath, The short story subtest has a slightly different format in which participants see the speaker saying a short story and are then asked two questions about it. They answer each question by choosing the correct picture from an array of four. For example one of the questions is “where is Ben going?” and the correct answer “school” is depicted along with three viable distractors “home”, “cinema” and “library”. Full details about the ToCS design, item selection and development can be found in Kyle et al. (2013). The instructions were specifically designed so that they could be delivered in the child's preferred communication or language, BSL, spoken English or a combination of the two. ToCs has been shown to have high external validity as an assessment of silent speechreading and good internal reliability (*α* = .80) (Kyle et al., 2013). The task took about 20 min to administer.

#### 2.2.3 Expressive vocabulary

The Expressive One Word Picture Vocabulary Test II (EOWPVT II: Brownell, 2000) was used to assess children's expressive vocabulary. Children are shown pictures of increasing difficulty and asked to name them. Children were allowed to respond in their preferred communication and therefore this task was providing an indication of their expressive vocabulary regardless of language preference. However, it should be noted that this task was designed to test English vocabulary and not BSL or sign language vocabulary. Following guidelines from Connor and Zwolan (2004), any answer that was not gestural was accepted. Two items in the test were changed to make it more suitable for British children, following Johnson and Goswami (2010) who used this test with deaf children in the UK. The item *racoon* was changed to *badger* and *the map of USA* to a *map of the UK*. In addition, and following pilot studies, we changed the pictures for two items to pictures more characteristic of British responses: *prescription* and *windmill*.

#### 2.2.4 Non-verbal skills

An estimate of non-verbal intelligence (NVIQ) was derived from the Matrices subtest of the British Abilities Scales II (BAS II: Elliot, Smith, & McCulloch, 1996). This test has been used previously with deaf children of similar age to those in the current study (see Harris and Moreno, 2004, Kyle and Harris, 2010).

### 2.3 Procedure

Children were all tested individually in a quiet room, normally adjacent to the classroom. Each child was seen over two testing sessions, not lasting more than 20 min each. All standardised tests were administered according to the instruction manuals but the instructions were delivered in the child's preferred communication method. Written parental consent was given for all children and the child's assent was also sought at the beginning of the first testing session. Ethical clearance was granted from the University Research Ethics Committee.

## 3 Results

### 3.1 Performance on the ToCS, vocabulary and reading tasks

The means and standard deviations for all tasks are presented in Table 2. Raw scores were used in all analyses as we were unable to obtain standard scores for all participants across all tests as a few of the children were just outside the top age range for the reading assessment. Standard scores are reported for the vocabulary assessment for descriptive purposes only. As a group, the hearing children achieved age-appropriate scores for reading accuracy and reading comprehension (mean chronological age = 9:01; mean accuracy reading age = 10:00; mean comprehension reading age = 9:08). The deaf children exhibited an average reading delay of sixteen months in reading accuracy and 22 months in reading comprehension (mean chronological age = 9:06; mean accuracy reading age = 8:02; mean comprehension reading age = 7:08). The hearing children had significantly higher vocabulary standard scores than the deaf children, *t*(175) = −10.87, *p* < .001, 95% CI −28.8 to −19.8. The mean vocabulary standard score for the deaf children was 76 (SD = 15.1) whereas the mean standard score for the hearing children was 100.2 (SD = 15.1). As reported in Kyle et al. (2013), deaf and hearing children did not differ in their speechreading skills (mean 49.0% vs. 50.6%, respectively) and showed an almost identical pattern of performance across the subtests. A two-way mixed design ANOVA (hearing status by ToCS subtest) revealed no statistically significant differences between the deaf and hearing children in their overall performance on ToCS, *F*(1,172) = .11, ns. There was a main effect of subtest, whereby children achieved higher scores on the single words > sentences > stories, *F*(2,344) = 294.61, *p* < .001. There was no significant interaction between group and ToCS subtest *F*(2,344) = .29, ns.

### 3.2 Correlations between reading, speechreading and vocabulary

Table 3, Table 4 present the partial correlations between reading, speechreading and vocabulary controlling for age and NVIQ. Age was statistically controlled, as performance on ToCS has been found to improve significantly with age over this age-range (see Kyle et al., 2013). NVIQ was also controlled for as although there was no significant association between NVIQ and speechreading, there were small yet significant associations between NVIQ and reading and vocabulary.

After statistically controlling for age and NVIQ, performance on ToCS (combined score across three subtests) for both deaf and hearing children was significantly related to reading accuracy (*r* = .49, *p* < .001 and *r* = .31, *p* = .005, respectively) and reading comprehension (*r* = .44, *p* < .001 and *r* = .28, *p* = .010). Performance on all three speechreading subtests was related to reading accuracy in deaf children; however only performance on the sentences was related to reading in the hearing children. Speechreading was also significantly associated with vocabulary knowledge (even after controlling for age and NVIQ) in both deaf children (*r* = .25, *p* = .021) and hearing children (*r* = .25, *p* = .02).

### 3.3 Multiple regression analyses

The main research aim was to determine the relative contributions of vocabulary and speechreading to reading ability in deaf children. A set of fixed order multiple regression analyses was conducted (see Table 5) to investigate whether speechreading and vocabulary were independent predictors of reading accuracy and comprehension. After controlling for age and NVIQ (entered in steps 1 and 2 and accounting for 54% of the variance), speechreading (entered in Step 3) accounted for 11% of the variance in the deaf children's reading accuracy scores. When vocabulary was entered in step 4, it accounted for an additional 13%. When the order in which they were entered into the regression analyses was exchanged so that vocabulary was entered in Step 3 before speechreading, it accounted for 15%. Speechreading still accounted for a small yet significant proportion of the variance (8%) in reading accuracy even when entered after vocabulary. Thus, speechreading and vocabulary seem to be relatively independent predictors of reading accuracy in deaf children as the proportion of variance each skill explains is not particularly dependent upon the order in which it is entered into the analysis.

The same analysis was conducted with reading comprehension as the dependent variable. Table 5 shows that speechreading and vocabulary were also independent predictors of reading comprehension for deaf children as the proportion of variance that each accounted for was fairly consistent regardless of the order in which they were entered. Age and NVIQ accounted for 59% of the variance in deaf children's reading comprehension scores. When entered in Step 3, speechreading accounted for almost 8% and vocabulary accounted for an additional 15% of the variance (in step 4). When the order in which vocabulary and speechreading was entered was switched, vocabulary accounted for 17% (step 3) and speechreading for 6% (step 4).

The same analyses were conducted for the hearing children (see Table 5). After controlling for age and NVIQ (68%), speechreading and vocabulary were both small yet significant predictors of reading accuracy, regardless of the order in which they were entered. Speechreading accounted for 3% (in step 3) and vocabulary accounted for an additional 3% (in step 4). If the order was switched, vocabulary accounted for 4% (in step 3) and speechreading accounted for 2% (in step 4). Therefore, vocabulary and speechreading are accounting for a portion of independent variance in reading accuracy scores in hearing children.

A different picture was observed when reading comprehension was the outcome variable for the hearing children. In this instance, speechreading was only a significant predictor if entered before vocabulary. Age and NVIQ accounted for almost 73% of the variance in reading comprehension so there was little variance left that could be accounted for. When entered in step 3, speechreading was a small predictor (2%) and vocabulary (step 4) accounted for 8%. However if the order was changed, vocabulary accounted for 9% but speechreading no longer accounted for any significant variance. Therefore, speechreading and vocabulary were small yet significant independent predictors of reading accuracy for hearing children, but in contrast to the deaf children, speechreading was not a significant independent predictor of reading comprehension.

### 3.4 How are background and audiological factors related to speechreading?

As speechreading was a strong predictor of reading in deaf children, the role of background and audiological factors in determining what makes a good child speechreader was examined. The effects of gender and NVIQ were investigated for both deaf and hearing children. A two-way ANOVA (gender × hearing status) revealed no effect of gender, *F*(1,176) = 3.47, ns, no main effect of hearing status (deaf vs hearing), *F*(1,176) = 3.47, ns, and no significant interaction, *F*(1, 176) = 1.44, ns. There was no significant association between NVIQ and performance on ToCS for deaf or hearing children (*r* = −.01, ns and *r* = −.06, ns respectively).

#### 3.4.1 Effect of hearing/audiological factors (degree of loss, type of hearing aid and age of diagnosis)

An additional set of analyses was undertaken for the deaf cohort. There was no significant correlation between degree of hearing loss and overall speechreading ability, *r* = −.17, ns. However, there was a significant negative correlation between degree of hearing loss and performance on the sentences subtest (*r* = −.25, *p* = .024) and stories subtest (*r* = −.27, *p* = .015) but not single words (*r* = .05, ns). Children with less severe levels of hearing loss scored higher on the sentences and the stories section. There was no effect of age of diagnosis on speechreading scores, *r* = .13, ns and there were no differences between deaf children with hearing aids (*n* = 49) and those with CIs (*n* = 35), *t*(81) = −.87, ns.

#### 3.4.2 Effect of communication preference

A two-way ANOVA revealed a main effect of child's communication preference, *F*(1,81) = 27.67, *p* < .001), whereby those children who preferred to communicate through oral language achieved higher scores on the ToCS than those who preferred to communicate through signing or total communication. There was also a main effect of speechreading subsection *F*(2, 162) = 178.16, *p* < .001) but no significant interaction *F*(2, 162) = 1.67, ns). Those children who preferred to communicate through oral language had lower levels of hearing loss than those who signed or used total communication, *t*(82) = −3.25, *p* = .002.

## 4 Discussion

The main aim of the current study was to investigate the relative contributions of vocabulary and speechreading to reading development and determine if these relationships held across different psycholinguistic levels. Speechreading and vocabulary were found to be independent predictors of reading ability in children but the precise strength of the relationship was dependent upon hearing status and the component of reading being investigated. For deaf children, speechreading and vocabulary were independent predictors of both reading accuracy and reading comprehension and accounted for an appreciable portion of the variance in their reading scores. In contrast, for hearing children, while both speechreading and vocabulary were independent predictors of reading accuracy, only vocabulary was an independent predictor of reading comprehension and accounted for a smaller proportion of the variance. These findings provide further evidence of the importance of speechreading and vocabulary for deaf children's reading (e.g. Kyle and Harris, 2010, Kyle and Harris, 2011, Easterbrooks et al., 2008, Mayberry et al., 2011) and for the idea that speechreading skills should not be ruled out as a factor influencing hearing children's reading (see Kyle & Harris, 2011). Due to the large age range in the current study, age understandably explained a lot of the variance in reading accuracy and reading comprehension scores which left little for speechreading and vocabulary to be able to explain. Thus, after age and NVIQ were entered into the analyses, it is remarkable that speechreading and vocabulary were indeed able to account for any further variance, particularly for the hearing children.

While it might be reasonable to expect a relationship between reading and speechreading in deaf children (where speechreading can provide access to phonological information in the absence of auditory input), it is not as immediately obvious why a small yet significant relationship would be observed for hearing children. However, the contribution of speechreading and vocabulary for hearing children can also be interpreted through the Simple View of Reading framework whereby vocabulary again provides input for the linguistic component while speechreading feeds into the decoding component. It is well-known that speech processing plays a role in the development of phonological skills and helps form phonological representations. What differs between the role of speechreading for deaf and hearing children's reading in this model is the necessity: for deaf children, speechreading is often one of the main ways of accessing spoken language whereas for hearing children, it is supplementary. The better a child is at speechreading, the more distinct their phonological representations are likely to be and more specified representations are linked with better reading (e.g. Elbro, 1996). This fits in with the viewpoint that a phonological code is not necessarily tied to the auditory domain but is abstract and therefore it can be derived from speech and speechreading (see Alegria, 1996, Campbell, 1997, Dodd, 1987). Information derived through speechreading has been shown to be processed in a similar manner to auditory speech (Campbell and Dodd, 1980, Dodd et al., 1983).

In prior research, the strong association reported between speechreading and reading in deaf children has been mainly limited to the level of word reading (Kyle and Harris, 2010, Kyle and Harris, 2011) whereas the current study extends this relationship to reading comprehension. However, previous studies only assessed speechreading of single words and the current study measured speechreading of words, sentences and stories; and a composite of these three levels was found to predict reading comprehension. It is also important to note that the age range in the current study was from 5 to 14 years whereas it was only 7–10 year olds in Kyle and Harris (2010) and therefore it is possible that speechreading plays a more important role in deaf reading comprehension as reading skills develop. This is in line with results from the deaf adult literature where speechreading has been found to correlate significantly with reading comprehension (Bernstein et al., 1998, Mohammed et al., 2006).

The extension of this association between reading and speechreading beyond single words is important as it suggests that the relationship is not due simply to perceptual matching (i.e. matching a single word token to a single speechread token). It is more likely to have a linguistic basis, especially as the ToCS was shown to be an ecologically valid assessment of speechreading (see Kyle et al., 2013). Speechreading of sentences and short stories requires higher-order linguistic skills such as parsing and grammatical knowledge, which are equally required for comprehension of written texts. It is important to remember that for deaf children, performance on all three psycholinguistic levels was related to reading accuracy and comprehension.

For both deaf and hearing children, vocabulary knowledge was the strongest independent predictor of reading accuracy and reading comprehension. This concurs with previous findings that language skills, including vocabulary knowledge, typically exhibit the strongest relationship with reading in deaf children (e.g. Easterbrooks et al., 2008, Kyle and Harris, 2006, Kyle and Harris, 2010, Kyle and Harris, 2011, Mayberry et al., 2011, Moores and Sweet, 1990, Waters and Doehring, 1990). Whilst this also fits with findings with hearing children, the exact strength of the relationship observed with hearing children usually depends upon which components of language and reading are being measured and the age of the children (see Ricketts et al., 2007). Future research should attempt to explore the contribution of broader language skills to reading development in deaf children rather than just vocabulary knowledge. It would also be interesting to determine whether the role of speechreading and vocabulary in deaf children's reading development is constant across different subgroups of deaf children.

Although speechreading and vocabulary were independent predictors of reading ability, they were also inter-related to some extent in both deaf and hearing children, as has been reported by Davies et al. (2009) for young hearing children. One interpretation of this relationship is that one cannot speechread a word that is not already in one's vocabulary; however, for deaf children it is equally plausible to suggest that speechreading leads to vocabulary growth and indeed for some it may be the only way that spoken words enter the mental lexicon. For deaf children in particular, it is most likely to be a reciprocal relationship rather than uni-directional. Another explanation can be found in the theories of Metsala and Walley (1998) and Goswami (2001) who argue that the development of phonological awareness and vocabulary are closely linked because as vocabulary knowledge expands, there is increased pressure for the underlying phonological representations to become more distinctive, which in turn leads to improved phonological awareness.

It is noteworthy that there were very few relationships observed between speechreading and other background and demographic skills. Similar to findings with deaf adults, those deaf children who preferred to communicate through speech were better speechreaders (see Bernstein et al., 1998). The lack of a significant effect of gender or NVIQ on child speechreading proficiency also concurs with more recent adult investigations (Auer & Bernstein, 2007). Although there was no overall association between degree of hearing loss and speechreading, deaf children with less severe levels of hearing loss scored higher on the sentences and the stories sections, perhaps suggesting a more supplementary functional use. It is reasonable to assume that speechreading combined with higher levels of residual hearing might lead to better speech perception (both audio-visual and visual alone) than lower levels of residual hearing combined with speechreading, although equally, the greater the level of deafness the more reliance one may have to place upon speechreading. This does raise a possible question over the validity of focusing on speechreading if it cannot be determined what makes a good speechreader in children. However, a better way of approaching this issue would be to implement speechreading training to determine whether it is a skill that can be trained in children.

Finally it should be noted that the current study is only correlational and therefore causality cannot be inferred. However, there is no reason to assume that the direction of the relationships observed in this study is any different to that reported in recent longitudinal studies (see Kyle and Harris, 2010, Kyle and Harris, 2011) in which speechreading and vocabulary predicted development in reading rather than reading predicting growth of speechreading and vocabulary.

## 5 Conclusions and educational implications

In conclusion, vocabulary and speechreading have been shown to be independent predictors of reading ability in deaf children and to a lesser extent in hearing children. It is likely that better speechreading skills result in more accurate phonological representations and the current results can be understood within reading models that suggest skilled reading necessitates both a decoding (speechreading) and a linguistic component (vocabulary). These findings suggest that focussing on both vocabulary development and speechreading skills in young deaf children may form a fruitful basis for helping support early reading development in young deaf children.

There are several educational implications that can be drawn from these findings. Teachers working with deaf children who use speech to communicate are likely to already have an understanding of the importance of visual speech and highlight this as a source of information. However our results show that speechreading is important for reading development in deaf children from other language backgrounds, including signing and possibly those with cochlear implants. Thus teachers working with these cohorts should also draw children's attention to the complementary phonological information that is visible on the face. Teachers and educators working with typically-developing hearing children should be aware that their children are also probably incorporating phonological information derived from visual speech into their representations and that encouraging children to be look at the lips when learning sounds is likely to help them form more distinct phonological representations. Drawing attention to information from visual speech is likely to not only help children initially distinguish between similar sounding phonemes but this information is likely to help create more distinct representations which will help with reading skills. The findings also provide further evidence for teachers working with either deaf or hearing children that reading is not only about decoding words, but that vocabulary, and most likely broader language skills not measured in the current study, also play an essential role in reading development.

## What this paper adds?

This paper investigates the importance of speechreading and vocabulary skills for reading development in a large cohort of deaf and hearing children ranging in age from 5 to 14 years. Previous research reported a relationship between speechreading and reading ability but only between speechreading of single words and word reading. The current study has extended this relationship to include larger units of speechreading (sentences and short stories) and to reading comprehension. Importantly, the current findings suggest that speechreading and vocabulary make independent contributions to reading ability in deaf children and possibly contribute to hearing children's reading. The results are interpreted within current theoretical frameworks of reading development for hearing and deaf children.

## Figures and Tables

**Table 1:** Descriptive statistics for deaf group by device use.

	Digital hearing aid *n* = 49	CI users *n* = 35	No device *n* = 2
Mean chronological age (*SD*)	9:04 (2:08)	9:06 (2:07)	10:08 (1:06)
Gender	24 boys	21 boys	2 boys
Mean hearing loss in db (*SD*)	92.4 (14.1)	104.3 (8.1)	110.0 (14.1)

Communication mode			
Speech (*n* = 44)	19	25	0
Signing (*n* = 33)	25	6	2
TC (*n* = 6)	3	3	0
Bilingual (*n* = 3)	2	1	0

Mean age of onset of deafness in months (SD)	17.8 (12.8)	16.0 (11.9)	15.5 (.7)
Mean NVIQ T Score (SD)	52.7 (7.7)	54.6 (6.9)	51.5 (3.5)

**Table 2:** Means (and SD) for performance on cognitive and language tasks.

	Deaf children *n* = 86	Hearing children *n* = 91
ToCS total (max = 40)	19.6 (6.5)	20.2 (6.1)
ToCS Words (max = 15)	9.7 (2.5)	9.9 (2.6)
ToCS Sentences (max = 15)	7.0 (3.3)	7.1 (3.5)
ToCS Stories (max = 10)	3.0 (1.6)	3.2 (1.3)
Vocabulary raw (max = 170)	62.3 (26.0)	87.1 (22.9)
Reading Accuracy raw (max = 100)	39.6 (24.1)	60.6 (27.4)
Reading Comprehension raw (max = 42)	12.1 (10.6)	22.4 (11.5)
NVIQ T score (*M* *=* 50, SD *=* 10)	53.4 (7.3)	55.5 (7.7)

**Table 3:** Partial correlations for Deaf children controlling for chronological age and NVIQ.

	ToCS total	ToCS Words	ToCS Sentences	ToCS Stories	Reading accuracy	Reading comprehension
Vocabulary	.25*	.25*	.14	.14	.58***	.64***
ToCS total	–	.75***	.87***	.70***	.49***	.44***
ToCS Words		–	.43***	.31**	.35**	.32**
ToCS Sentences			–	.49***	.44***	.37**
ToCS Stories				–	.28*	.30*
Reading accuracy					–	.83***

* *p* < .05.

** *p* < .01.

*** *p* < .001.

**Table 4:** Partial correlations for Hearing children controlling for chronological age and NVIQ.

	ToCS total	ToCS Words	ToCS Sentences	ToCS Stories	Reading accuracy	Reading comprehension
Vocabulary	.23*	.18	.25*	.02	.38***	.58***
ToCS total	–	.81***	.87***	.42***	.28*	.26*
ToCS Words		–	.52***	.10	.15	.12
ToCS Sentences			–	.21	.35**	.29*
ToCS Stories				–	.05	.14
Reading accuracy					–	.76***

* *p* < .05.

** *p* < .01.

*** *p* < .001.

**Table 5:** Multiple regression analyses for deaf and hearing children.

		Deaf children				Hearing children			
Step	Independent variable	Reading accuracy		Reading comprehension		Reading accuracy		Reading comprehension	
		*R*^2^	*R*^2^ change	*R*^2^	*R*^2^ change	*R*^2^	*R*^2^ change	*R*^2^	*R*^2^ change
1	Age	.499	.499***	.500	.500***	.665	.665***	.708	.708***
2	NVIQ	.539	.040*	.592	.092***	.675	.010	.728	.020*
3	Speechreading	.643	.105***	.670	.078***	.705	.030**	.749	.021*
4	Vocabulary	.773	.129***	.818	.148***	.736	.031**	.825	.075***
3	Vocabulary	.691	.153***	.761	.169***	.720	.046***	.819	.091***
4	Speechreading	.773	.081***	.818	.057***	.736	.016*	.825	.006

* *p* < .05.

** *p* < .01.

*** *p* < .001.
